# Supplementary material for: Comprehensive Annotation and Functional Exploration of MicroRNAs in Lettuce
Source: Front Plant Sci. 2021 Dec 24;12:781836. doi: 10.3389/fpls.2021.781836 (PMC8739914; doi:10.3389/fpls.2021.781836)
Supplement: Supplementary file 3 [file Data_Sheet_9.ZIP › GSM2230751.plot/Lsa-miRN1690_Lsat_1_v5_gn_8_112140.1_351_TPlot.pdf]

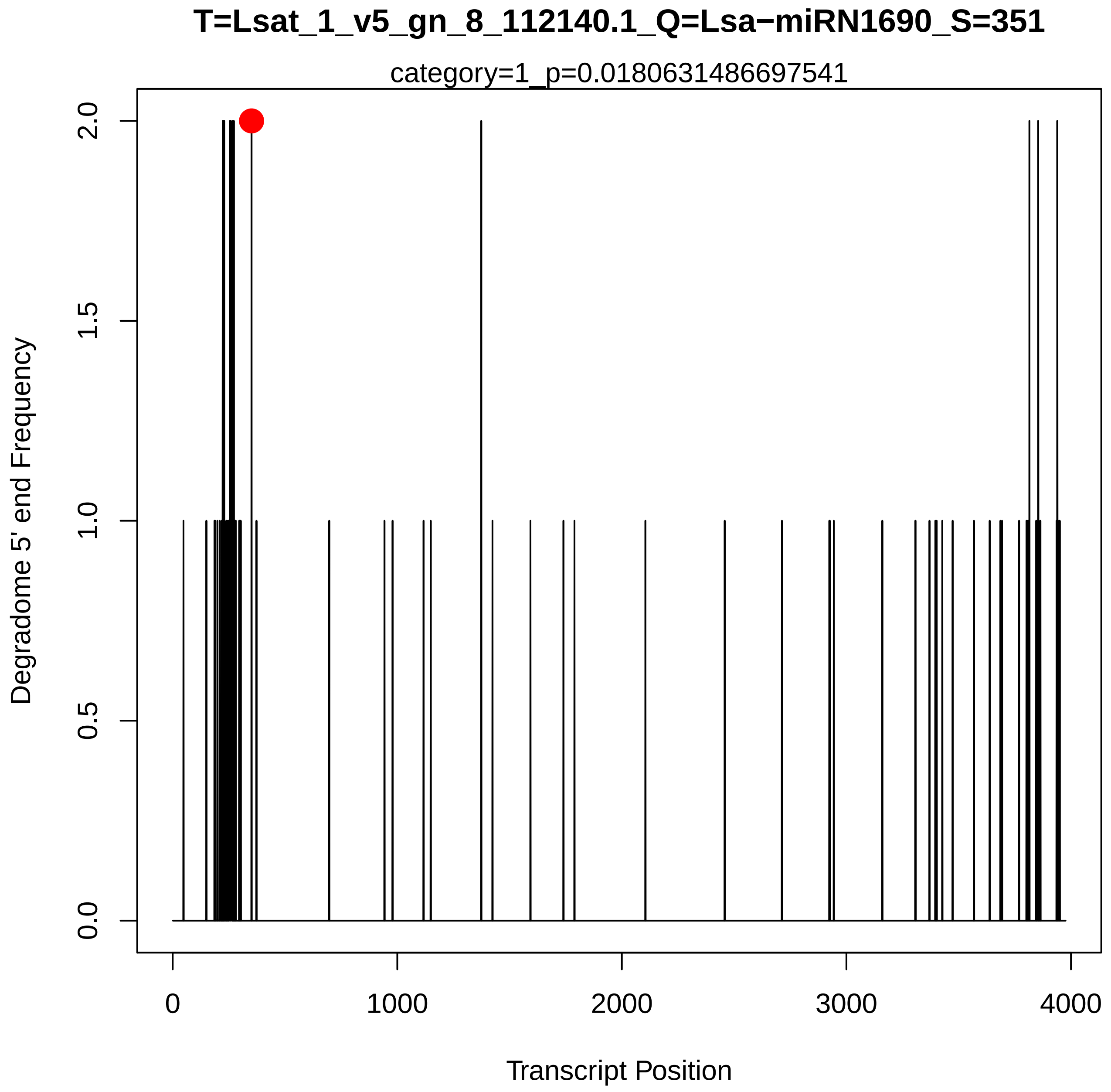

T=Lsat_1_v5_gn_8_112140.1_Q=Lsa−miRN1690_S=351
category=1_p=0.0180631486697541
Degradome 5' end Frequency
0.0
0.5
1.0
1.5
2.0
0
1000
2000
3000
4000
Transcript Position
